# Expanding the role of bacterial vaccines into life-course vaccination strategies and prevention of antimicrobial-resistant infections

**DOI:** 10.1038/s41541-020-00232-0

**Published:** 2020-09-11

**Authors:** Jan T. Poolman

**Affiliations:** Bacterial Vaccine Discovery & Early Development, Janssen, Leiden, Netherlands

**Keywords:** Bacterial infection, Vaccines

## Abstract

A crisis in bacterial infections looms as ageing populations, increasing rates of bacteraemia and healthcare-associated infections converge with increasing antimicrobial resistance and a paucity of new antimicrobial classes. New initiatives are needed to develop bacterial vaccines for older adults in whom immune senescence plays a critical role. Novel vaccines require an expanded repertoire to prevent mucosal diseases such as pneumonia, skin and soft tissue infections and urinary tract infections that are major causes of morbidity and mortality in the elderly, and key drivers of antimicrobial resistance. This review considers the challenges inherent to the prevention of bacterial diseases, particularly mucosal infections caused by major priority bacterial pathogens against which current vaccines are sub-optimal. It has become clear that prevention of many lung, urinary tract and skin infections requires more than circulating antibodies. Induction of Th1/Th17 cellular responses with tissue-resident memory (Trm) cells homing to mucosal tissues may be a pre-requisite for success.

## Bacterial vaccines: where do we stand?

One of the greatest public health challenges of the 21st century is the prevention and treatment of bacterial infections. A confluence of ageing populations at high risk of infection, increasing antimicrobial resistance (AMR), increasing global morbidity and mortality due to bacteraemia and healthcare-associated infections, combined with an absence of new antimicrobial classes, heralds a crisis in bacterial diseases^1^. Bacterial vaccines can have positive impacts on AMR at multiple levels. Vaccines can prevent community- and healthcare-associated infections with multi-drug-resistant (MDR) pathogens, directly reduce antimicrobial consumption and development of resistance, and prevent infections in all age-groups; particularly in older adults who have emerged as the age-group with the highest unmet vaccine needs^2–5^.

Vaccines were originally developed to prevent potentially lethal childhood infections. Relatively uncomplicated techniques allowed early development and production of effective toxoid vaccines against diphtheria and tetanus, and live attenuated or killed whole-cell vaccines against whooping cough, cholera, tuberculosis and typhoid. Later vaccines were built on immunoprotective subunits, such as acellular pertussis (aP) vaccines, vaccines targeting Lyme disease, plain polysaccharides (such as 23-valent pneumococcal polysaccharide vaccine), and capsular polysaccharides covalently bound to a carrier protein (conjugate vaccines targeting; *Streptococcus pneumoniae*, *Neisseria meningitidis*, *Haemophilus influenzae* type b [Hib] and most recently, *Salmonella enterica* serovar Typhi). More recently, in silico antigen discovery and/or subtractive antibody-screening was used to develop *N. meningitidis* serogroup B (MenB) subunit vaccines^6^.

In the 21st century the focus of vaccine development has moved from childhood infections to preventing infections that occur throughout all life stages. This focus includes improving currently available vaccines to increase their efficacy at the extremes of age (such as pertussis in neonates and pneumococcal pneumonia in seniors), or to increase the duration of efficacy (such as pertussis vaccines), and developing new vaccines targeting pathogens of global significance, such as *Staphylococcus aureus*, *Escherichia coli*, *Klebsiella pneumoniae*, *Mycobacterium tuberculosis* (Mtb), *S. enterica* serovar Typhi*, Chlamydia trachomatis*, *S. pyogenes*, *N. gonorrhoeae, Clostridioides* (previously *Clostridium) difficile*, and many others^7^. This review summarises where we stand with current vaccine efforts focusing on major priority bacterial pathogens. To be successful, new or improved bacterial vaccines need to broaden their scope beyond toxin neutralisation and prevention of invasive disease, to prevention of mucosal diseases such as tuberculosis, skin and soft tissue infections caused by *S. aureus*, urinary tract infections (UTI) caused by *E. coli*, and pneumonia caused by *S. pneumoniae*. Particular focus is given to the needs of older adults who are at high risk of severe bacterial diseases such as pneumonia and bacteraemia. Older adults are a previously neglected group in whom bacterial diseases are currently responsible for more morbidity and mortality that all other age-groups^5^. Overcoming the specific immunological challenges associated with the development of vaccines that are effective in older persons is a significant hurdle and has become a strong driver of vaccine design. While new bacterial vaccines have the potential to provide benefits to all age-groups, it is the population of ageing persons who could reap the greatest benefits from improved disease prevention. This means that new bacterial vaccines are likely to be developed first for older persons, and then extended to other age-groups and special populations, such as immunocompromised persons, in a later step. An example of such an approach has been the zoster adjuvanted subunit vaccine^8^.

We also consider pathogens with high levels of MDR, many or most of which are also important pathogens in older adults. Preventing mucosal infections will require new vaccine strategies and the challenges specific to important mucosal diseases caused by MDR pathogens of global significance are discussed.

## Antimicrobial resistance

**Fig. 1:**
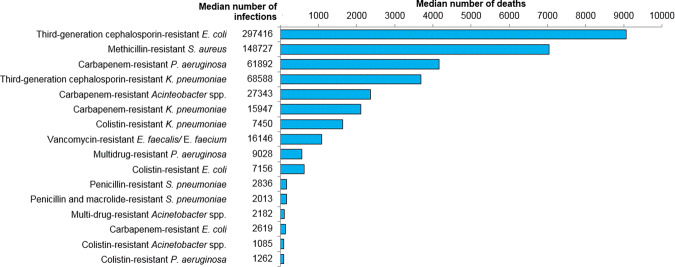
Deaths due to antimicrobial-resistant pathogens using 2015 data from the European Antimicrobial Resistance Surveillance Network (EARS-Net)^10^. The burden of disease and death was modelled using 2015 data from the European Antimicrobial Resistance Surveillance Network (EARS-Net) country-corrected for population coverage. The graph shows the median estimated number of infections and deaths caused by 16 antibiotic-resistant pathogens of public health importance.

The World Health Organization (WHO) recognises AMR as one of the top 10 threats to human health^9^. In Europe, 63.5% of AMR infections were healthcare-associated in 2015^10^, and adults aged 65+ years were the second-most affected group after infants. It is estimated that each year 700,000 deaths are linked to AMR globally^1^. The pathogens commonly implicated in AMR infections are those collectively referred to as ESKAPE pathogens: *Enterococcu faecium*, *S. aureus*, *K. pneumoniae, Acinetobacter baumannii*, *P. aeruginosa*, and *Enterobacter* spp^11^. Critically, *E. coli* is absent from this list despite being the most common cause of healthcare associated infections and the most common cause of death due to AMR infection (Fig. 1)^10,12^. *E. coli* is underappreciated in terms of its clinical importance, as reflected by its exclusion from the ESKAPE acronym and from surveillance networks of invasive bacterial disease, such as the US Center for Disease Control and Prevention’s ABC surveillance system^13^, even though it is the number one cause of such invasive diseases^14,15^. The tremendous diversity in clinical pathogenicity w ithin the *E. coli* species may have contributed to *E. coli* being overlooked. The introduction of different etiological names that more accurately distinguish between toxin-producing diarrhoeagenic *E. coli*, and *E. coli* that causes UTI and bacteraemia/sepsis, as historically achieved for *Shigella* species, may clarify the clinical disease burden attributable to different pathotypes. Extra-intestinal Pathogenic *E. coli* (ExPEC) stands out as the leading cause of UTI, healthcare-associated infections and bacteraemia, and is the leading antimicrobial-resistant pathogen, exemplified by ExPEC strains that produce extended-spectrum-beta-lactamases (ESBL)^16^.

In 2017, the WHO published a list of priority AMR pathogens to direct research and development of effective treatments^7^. Priority 1 bacteria are carbapenem-resistant *A. baumannii*, carbapenem-resistant *P. aeruginosa*, and carbapenem- and third generation cephalosporin-resistant (ESBL) Enterobacteriaceae (predominantly ExPEC and *K. pneumoniae*), with methicillin-resistant *S. aureus* (MRSA)/vancomycin-resistant *S. aureus* ranked as a Priority 2 pathogen^7^. Outside of this list lies Mtb, which caused 1.6 million deaths in 2017^17^. MDR Mtb strains caused 3.5% of new tuberculosis cases and 19% of existing cases, of which 8.5% were extensively drug resistant^17^. Globally, tuberculosis treatment success using antimicrobials is low (55%)^17^, and better antimicrobials as well as vaccines that prevent infection and vaccines that prevent disease are urgently needed.

*C. difficile* is another priority pathogen which has not acquired AMR, but which is usually directly associated with antimicrobial-induced changes to the microbiome. *C. difficile* causes an estimated 29,300 deaths annually in the US, the vast majority in patients aged 65+ years^18,19^. Almost all *C. difficile* infections occur in the context of a healthcare event and antimicrobial use.

Drug-resistant *Candida* spp (a fungus) is also identified as a significant antimicrobial-resistant threat^20^. *Candida* spp is a leading pathogen in hospitalised patients with sepsis and septic shock, with an incidence that is similar to other major pathogens such as *K. pneumoniae* and *Pseudomonas* spp^21^. Without a solution to emerging AMR, global deaths due to AMR infections are predicted to reach 10 million annually by 2050, at an economic cost of 2–3.5% of global gross domestic product^1^. Modelling suggests that *E. coli* resistance accounts for almost half of the economic impact resulting from AMR infections^1^. Vaccines demonstrably prevent MDR infections and reduce antimicrobial consumption, but their potential value in the fight against AMR and their role in an integrated AMR strategy that includes targeted antimicrobials and improved diagnostics is as yet unrealised^2,22,23^.

## The vaccine needs of seniors

**Fig. 2:**
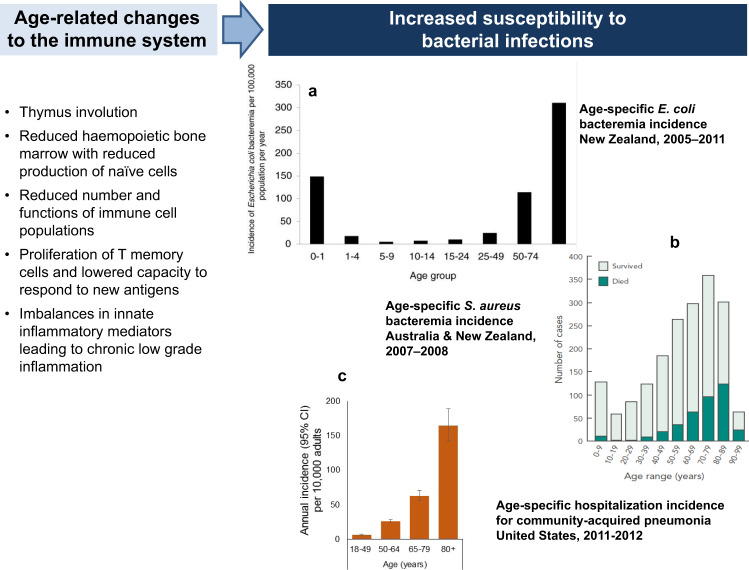
Age and infection: immune senescence leads to increased susceptibility to bacterial infections. **a** The age-specific incidence of *E. coli* bacteraemia in all age-groups and highlights the markedly increased disease burden after age 50 years. **b** The number of cases of *S. aureus* bacteraemia with higher case numbers in older adults. The proportion of patients who died also increased with age. **c** The incidence of hospitalised community-acquired pneumonia in adults in the US, which increases substantially with age. Insert **a** reproduced from Williamson et al.^126^. Insert **b** reproduced with permission from Turnidge et al.^127^. Data for insert **c** from Jain et al.^128^.

The proportion of individuals aged 65+ years is expected to reach 16% of the global population by 2050, and the health of the aged will have immense impacts on global productivity and consumption of healthcare resources^24^. The functional capacity of all aspects of the immune system decreases as part of the natural ageing process (immune senescence) (Fig. 2)^25^. As a result, older adults are at increased risk of infection and have reduced capacity to respond to vaccination (Fig. 2). Compared to younger age-groups, seniors experience higher rates of gastroenteritis, UTI, skin and soft tissue infections, pneumonia and bacteraemia/septicaemia^26^, and pneumonia and septicaemia rank as the most common causes of infectious disease-related deaths among older adults in the United States^27^. The leading causes of community-onset and healthcare-related bacteraemia/septicaemia are ExPEC and *S. aureus*^15,28–32^. In the US, *E.coli* was identified in 33.7% of cases of culture-positive community-onset sepsis, followed by *S. aureus* in 21.3%^32^. Together, ExPEC and *S. aureus* are responsible for approximately 50% of all bacteraemia/septicaemia cases^14,33^, are the commonest causes of antimicrobial AMR, and cause more deaths due to AMR infections than any other pathogen (Fig. 1). Up to 64% of bacteraemia and 70% of deaths due to bacteraemia occur in adults aged 65+ years^14,34,35^.

Seniors are the second highest consumers of antimicrobials after young children, accounting for 38.5% of all antimicrobial prescriptions^26^, and are therefore key drivers of AMR. Currently available bacterial vaccines have proved unequal to the task of protecting older adults because they are unable to overcome the challenges of immune senescence, and the effectiveness of vaccine-based disease prevention strategies in older adults has only been modest.

A fundamental change in the senior vaccination landscape occurred with the licensure of the recombinant herpes zoster adjuvanted subunit vaccine (RZV, *Shingrix™*, GSK) that showed 91% efficacy in preventing herpes zoster in adults aged 80+ years^36^. The unprecedented efficacy of RZV in older adults is likely due to the use of the AS01 adjuvant (*Quillaja saponaria* Molina: fraction 21 and 3-deacylated monophosphoryl lipid A presented as liposomes), and showed for the first time that older adults retain the capacity to respond effectively to vaccination given the right stimulus. Subsequently, a hepatitis B vaccine licensed in the US containing CpG, a TLR9 agonist (*Heplilav-B*, Dynavax), also showed improved immunogenicity in older persons; 91.6% seroprotection in 60–70 year olds compared to 72.6% following an alum-adjuvanted hepatitis B vaccine^37^. Whether these or other adjuvants can be equally successful in preventing bacterial infections is under investigation. If successful, novel adjuvants could herald a new era of highly effective vaccine development for the globally significant population of older adults, with positive flow-on effects in reducing rates of antimicrobial consumption.

Outside of developed countries tuberculosis is a common cause of death in older adults. Globally, the incidence of new and relapsed tuberculosis is high in adults aged 65+ years, particularly in the Eastern Mediterranean, China, South East Asia and the Western Pacific regions where the highest incidences of tuberculosis are in this age group^17,38^.

Critical bacterial vaccines are lacking for seniors, and in the same way that infant vaccination schedules comprise a bespoke package of highly effective vaccines that target infections that are either common, serious, or both, seniors warrant the same approach. The senior vaccination package targeting bacterial infections should ideally prevent disease caused by *S. pneumoniae, E. coli, S. aureus, C. difficile, K. pneumoniae* and Mtb. Probably the fungus *Candida* also qualifies as a target older age vaccine. Vaccines for each of these pathogens need to be tailored to the site of infection (bloodstream, skin, and mucosal surfaces of the respiratory, genito-urinary and gastrointestinal tracts) and be capable of overcoming the immune evasive mechanisms used by bacteria against the host response.

## Determinants of vaccine design

### Bacterial strategies of immune evasion

**Fig. 3:**
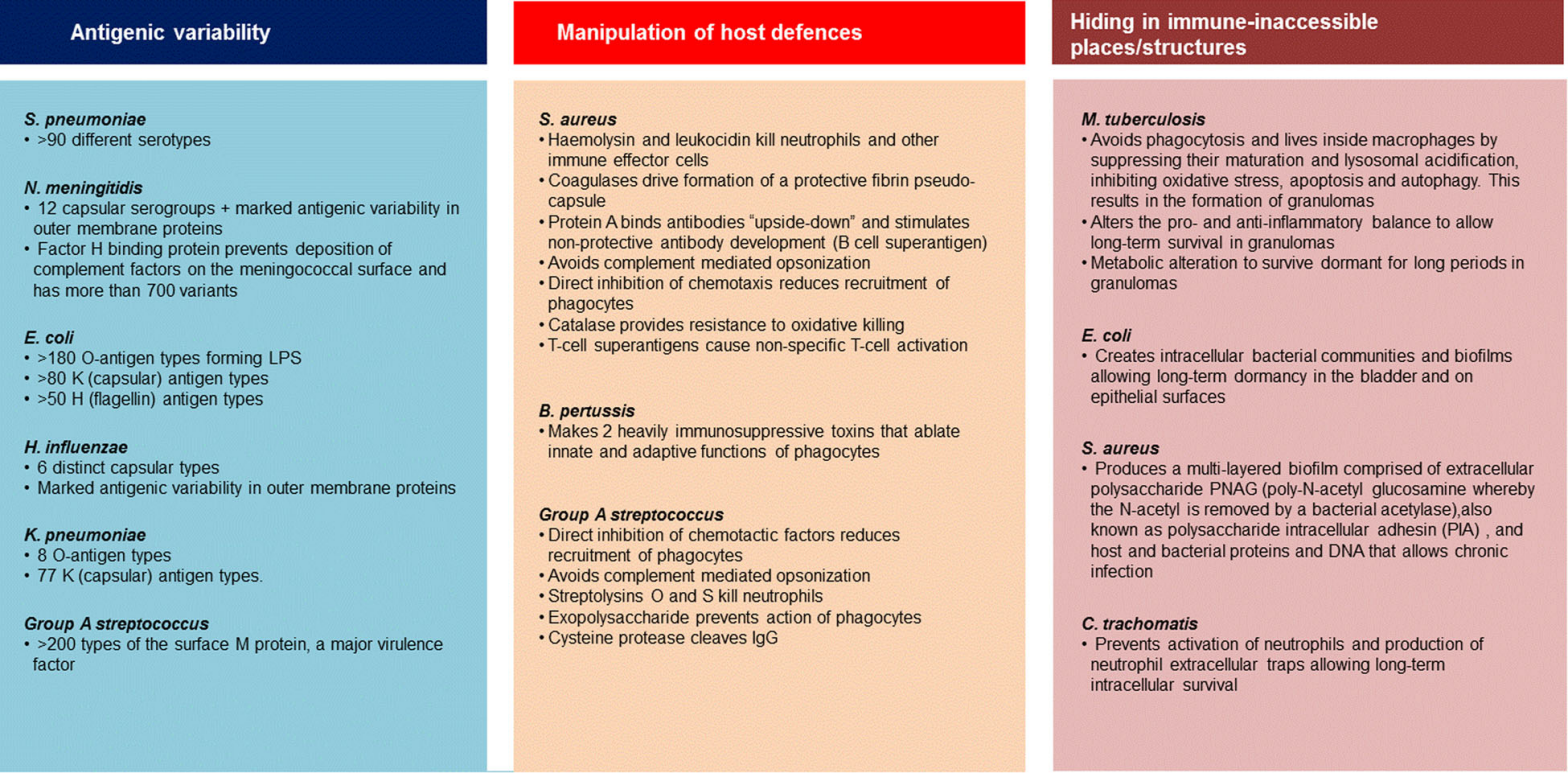
Mechanisms of immune evasion by bacterial pathogens. Immune evasion can be broadly grouped into three strategies. Examples summarise the important evasion mechanisms employed by representative pathogens, but do not include all possible potential mechanisms.

Bacteria use a vast array of physical, biochemical and immunological mechanisms to successfully colonise and infect the human host, while evading or neutralising attempts by the host to destroy them (Fig. 3)^39–41^. In the broadest sense, these strategies can be grouped as antigenic variability/heterogeneity, usually in surface polysaccharides and/or surface proteins such that type-specific immunity does not prevent subsequent infections with other types of the same bacterium; manipulation of the immune system whereby bacteria escape host killing by interrupting/inactivating host response strategies, or hiding in immune-inaccessible places/structures. These strategies influence vaccine design, potentially requiring the inclusion of multiple antigens/serotypes to maximise coverage, or the inclusion of immune-enhancing components such as adjuvants.

### Challenges associated with different infections sites: invasive versus mucosal diseases

**Fig. 4:**
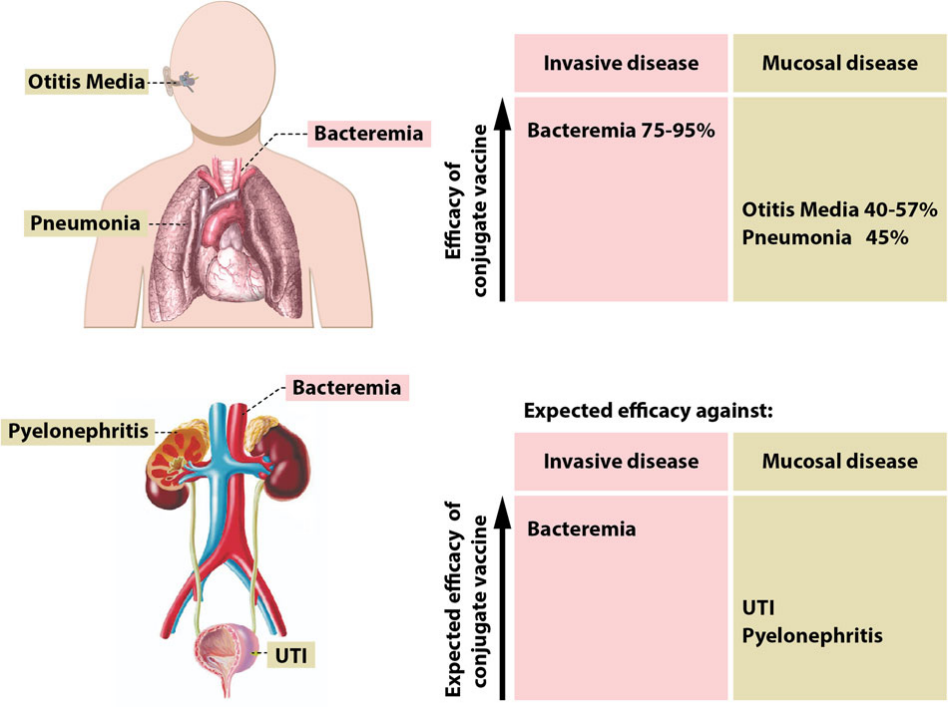
Efficacy conjugated vaccines lower in mucosal tissues compared to bacteraemia^44,129–132^. The top figure provides published estimates of pneumococcal conjugate vaccine efficacy against invasive and mucosal disease caused by *S. pneumoniae*. Efficacy is high against invasive disease but lower against mucosal diseases. The lower figure extrapolates these findings to what might be expected in terms of vaccine efficacy for an ExPEC conjugated vaccine. The image of respiratory system was made by Mikael Häggström and obtained from https://commons.wikimedia.org/wiki/File:Heart_and_lung.png. The image of the urinary tract was created by Javier Ramos Sancha and obtained from http://sgaguilarjramos.blogspot.com/2014/01/respiratory-system-and-excretory-system.html.

Invasive diseases such as meningitis and bacteraemia/septicaemia can be prevented by the presence of neutralising antibodies in serum. For many invasive diseases the protective level of antibody has been defined. Accordingly, Hib, meningococcal, and pneumococcal conjugate vaccines that use T-cell help to increase B-cell responses against the capsular polysaccharides, and increase antibody production and B memory cells, are very effective in inducing neutralising antibodies against vaccine-specific serotypes/serogroups and demonstrate very high efficacy (at least 75%) in preventing invasive disease^42,43^. In marked contrast, the efficacy of these same vaccines against mucosal diseases (otitis media and pneumonia) is only intermediate at best (Fig. 4). For pneumococcal conjugate vaccines (PCVs), efficacy against pneumonia in infants and adults aged 65+years, and efficacy in preventing otitis media is up to 50% lower than for bacteraemia (Fig. 4)^44^. Efficacy with candidate *S. aureus* vaccines in preventing skin/surgical site infections has not yet been achieved, and a candidate ExPEC conjugate vaccine has demonstrated an early weak signal suggesting intermediate efficacy against UTI^45^. The reason for the less than optimal efficacy is because antibodies induced by parenteral vaccination have to make their way from serum to mucosal surfaces such as the middle ear, bladder epithelium, nasopharynx and cervix, by passive transudation^46–48^. Opsonization by phagocytes also requires the physical re-location of neutrophils onto the mucosal surface. Thus, while the majority of infectious pathogens gain entry to the human body via mucosal surfaces, vaccines that induce long-lived protective immune responses at the mucosal surface have been difficult to achieve. Induction of bystander CD4 T-cell immunity and Tissue-resident memory (Trm) homing to the mucosa, combined with attracting phagocytic cells to the mucosa, may hold the key.

### The induction of Th1/Th17 and their role in controlling mucosal infections

**Fig. 5:**
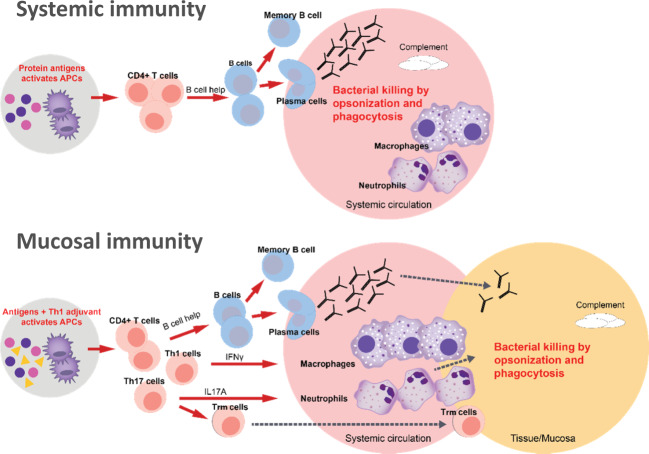
Differential CD4+ T-cell responses needed to achieve serum versus mucosal extracellular bacterial killing. Top panel: After vaccination, bacterial protein antigens reach draining lymph nodes and are taken up by dendritic cells that differentiate into active antigen-presenting cells (APCs). Activated CD4+ T helper cells release inflammatory mediators specific to the activated sub-population (Th1, Th2, Th17), facilitating amongst others the differentiation of B-cells into antibody-producing plasma cells or B-memory cells. In the systemic circulation, bacterial pathogens are readily exposed to circulating antibody and complement factors that coat the bacterial cell surface, leading to its recognition and destruction by phagocytic cells. Lower panel: The same process is enhanced by a Th1 adjuvant in the vaccine that preferentially activates the CD4+ Th1 and Th17 subfamily of T-cells. Th1 and Th17 cells release cytokines including IFNy and IL17A that activates and recruits macrophages and neutrophils that migrate to the mucosal surface. Antibodies produced by plasma cells transudate onto the mucosal surface at lower levels than achieved in the systemic circulation, and bystander T-cell immunity supports antibody-mediated opsonophagocytosis.

In addition to the critical role of the presence of opsonic antibodies at the mucosa, attracting phagocytic cells across the mucosa requires a Th1/Th17 immune response. If the mucosa is breached by pathogenic bacteria, CD4+Th1/Th17 T-cells are needed to attract and activate neutrophils and macrophages to clear extracellular pathogens and facilitate opsonic antibodies. Therefore, vaccines are needed that induce this type of response (Fig. 5)^49^. The importance of this T-cell bystander immunity in preventing infection (for example, pertussis, staphylococcal, and pneumococcal infections) has been demonstrated in animal models including non-human primates^50–52^.

Th1 and Th17 responses are induced by specific cytokines produced mainly by innate cells/antigen-presenting cells (APCs) upon TLR activation by microbial or parasitic compounds. More specifically, interferon-gamma (IFN-γ) and interleukin (IL)-12 are key players in the differentiation of naïve CD4+T-cells into Th1 cells that are characterised by high production of IFN-γ. The differentiation of naïve CD4+ T-cells into Th17 cells is brought about by transforming growth factor beta, IL-6 and IL-21 produced by APCs after stimulation by antigens from pathogens that cause mucosal diseases, such as *K. pneumoniae*, Mtb, *Helicobacter pylori*, and *B. pertussis* among others^53^. IL-23 stabilises the commitment of Th17 cells to this lineage. The transcription factor RORγt is a key regulator of the Th17 differentiation^54^.

Mucosal T-cells positioned at barrier surfaces are directed towards effector Th1 and Th17 responses. Th1 cells produce mainly IFN-γ, IL-2 and tumour necrosis factor alpha. IFN-γ is a key cytokine for the differentiation and activation of macrophages and dendritic cells leading to enhanced ability to kill intracellular pathogens such as *S. enterica* serovar Typhi and Mtb^55,56^. IL-17 primarily stimulates innate mucosal defences, particularly by activating neutrophils. Recent evidence also suggests a role for this cytokine in initiating the B-cell response by the preferential expression of B-cell chemoattractant CXCL13 on Th17 cells^57,58^.

Trm cells serve as the frontline defence at mucosal sites such as the urinary tract, vagina, lung and skin, and are in part derived from Th17 effector cells. For example, Trm are needed for bacterial clearance of antimicrobial-resistant *K. pneumoniae strains*. Interestingly, a percentage of lung resident Trm cells retain the ability to produce only IFN-γ, known to be important in controlling *K. pneumoniae* infection^59^.

Genetic deficiency of IL-17 receptor A abrogates responses to IL-17A and IL-17F, and is linked to chronic mucocutaneous candidiasis, staphylococcal skin diseases, and bacterial respiratory infections, suggesting a pathogen-specific role for IL-17RA^60^.

By themselves, Th1/Th17 responses alone are not sufficient to prevent mucosal infections^61^. Antibodies reach mucosal surfaces with greater ease than cells and are the main effectors of bacterial killing and clearance at the mucosa (Fig. 5). Bystander Th1/Th17 mechanisms are critical but bacterial killing requires the presence of opsonophagocytic antibodies from activated B cells, as elegantly demonstrated in a *B. pertussis* mouse model transferred with human immune cells^61^. Adjuvants that induce Th1/Th17 differentiation and IFNy/IL-17-production by innate cells are necessary to induce bystander T-cell immunity to support primarily antibody-mediated protective efficacy against mucosal infections. The recent demonstration that Th17 effector cells can develop into Trm cells further pinpoints the importance of this arm of the immune system for defences against *S. aureus*, *Candida* spp and respiratory bacterial pathogens. In practice however, little is yet known about vaccine-induced Th1/Th17 responses; either how they can be reliably induced, what defines the optimal balance between Th1 and Th17 responses to ensure protection without immune dysregulation, or the long-term consequences of Th17 induction. Evidence supporting that Th1/Th17 responses induced by vaccination can be beneficial include whole-cell (wP) vaccines that have a longer duration of protection than acellular pertussis (aP) vaccines, and which induce Th1/Th17-biased responses in infants in contrast to Th2-directed responses by aP^62^. In mice, wP and adjuvanted intranasal pertussis vaccines induced Th17 responses and showed higher efficacy against nasal infection than an aluminium-adjuvanted aP vaccine^63,64^. BCG revaccination of adults in India was also shown to boost anti-mycobacterial Th1/Th17 responses^65^. This, in combination with evidence that BCG revaccination prevented sustained seroconversion by IFN-γ release assay infection in a South African study^66^, may suggest an association between successful induction of Th1/Th17 and prevention of Mtb infection.

There is a fine balance that defines a protective versus pathological role for IL-17 at mucosal sites, and future studies are necessary to identify the cytokine milieu that induces IL-17 protective responses in response to vaccination to increase efficacy and avoid immune dysregulation (Box 1).

Box 1 IL-17, a two-edged sword IL-17 is a family of cytokines secreted by T helper cells that bind to specific IL-17 receptors on epithelial cells. The main source of IL-17 production is Th17 cells stimulated by IL-23 and IL-6 released from sub-epithelial antigen-presenting cells after exposure to a microbial pathogen. IL-17 can also be produced by innate lymphoid cells including ƴδ cells^133^.

Function: The Th17 response protects against extracellular bacterial pathogens in the lung, intestine and skin^133^. Th17 cells appear to naturally track to the mucosa and release IL-17 and IL-22 that act to upregulate the expression of antimicrobial peptides, and induce epithelial cells to express cytokines and chemokines (IL-8, CXCL1 and CXCL2, G-CSF and GM-CSF) that recruit tissue-infiltrating neutrophils to the mucosa. IL-17 also has a role in maintaining the structural integrity of the mucosal barrier and promotes regeneration of inflamed epithelial surfaces.

Dysregulation: IL-17 production is tightly regulated by Treg cells. Beyond the mucosa, dysregulation of the Th17 response has been implicated in the development of autoimmune disease such as psoriasis, asthma, rheumatoid arthritis, inflammatory bowel disease^134^.

Implications for vaccines: Induction of a transient Th17 response may be crucial for a successful vaccine targeting mucosal pathogens. Careful evaluation of IL-17 production and kinetics in the hours and days post-vaccination will help to guide optimal vaccine design.

## Developing new bacterial vaccines

### Polysaccharide-protein-conjugate vaccines

The technically complex protein-conjugate vaccines were not developed until the 1980s but have proven immensely successful. Poorly immunogenic surface polysaccharides are chemically linked (using oxidation and reductive amination as for PCV13, or cyanylation as for PCV10^67,68^) to a T-cell-dependent carrier protein. The glycoconjugate is taken up by antigen-presenting cells such as saccharide-specific B-cells which then display digested peptides on the cell surface. These peptides are presented by APCs in the context of MHCII molecules to T helper cells that induce the differentiation of the saccharide-specific B cells into anti-saccharide antibody producing plasma cells and B memory cells (Fig. 5). Licensed PCV, Hib and meningococcal conjugate vaccines are among the most successful bacterial vaccines. Since the implementation of PCVs in low-income countries by the Gavi Vaccine Alliance, vaccination is estimated to have prevented at least 500,000 childhood deaths, mainly from pneumonia^69^.

Temporal changes in the distribution of disease-causing serotypes (serotype replacement) have been observed after introduction of PCVs, although the overall effects on invasive disease incidence have so far been small^70–72^. Capsular switching caused by genetic recombination events occurs infrequently and independently of vaccine introduction^73^. PCV15/20/24 developments are ongoing that may improve control of serotype switching and might deliver the advantages of PCVs to seniors, replacing the 23-valent plain polysaccharide vaccine. However, current conjugate vaccines provide only intermediate efficacy against mucosal diseases and efficacy against adult pneumonia in particular, needs to be improved (Fig. 4)^44,74,75^. In this respect, novel adjuvants and mucosal (instead of parenteral) administration need to be investigated. For example, a pneumococcal protein-based vaccine using the AS02 adjuvant, a combination of immunostimulants (*Quillaja saponaria* Molina: fraction 21 and 3-deacylated monophosphoryl lipid A presented as an oil in water emulsion), protected non-human primates against pneumococcal pneumonia^76^.

Bioconjugation and synthetic/semi-synthetic saccharide synthesis are techniques with the potential to improve and expand the repertoire of second-generation conjugate vaccines. Bioconjugation refers to the biosynthesis of polysaccharide/s and carrier protein in parallel in *E. coli* cells, with in vivo coupling via oligosaccharyltransferases such as PglB^77,78^. A phase 1/2a clinical trial of a multivalent ExPEC bioconjugate vaccine in older adults is currently ongoing (NCT03819049). Invasive ExPEC disease (predominantly bacteraemia and urosepsis) is the leading cause of bacterial invasive disease in older adults and a vaccine to prevent this will likely be welcomed. A synthetically manufactured Hib vaccine has been used in the national immunisation programme in Cuba for more than a decade^79^. Non-covalent high-affinity conjugation using multiple antigen presentation systems further adds to the conjugate toolbox^80^.

In glycoconjugates, polysaccharides appear to have some capacity to activate T-cells when presented as targeted glycopeptides with the peptide part presented in the context of MHCII molecules, and the saccharide part stimulating T-cell responses in addition to, and on top of the saccharide-specific antibody responses^81^. This mechanism may open new avenues of conjugate vaccine development.

### Acellular pertussis vaccines: subunit vaccines

aP vaccines were the first bacterial protein subunit vaccines and they contain between 1 and 5 purified protein subunits. Despite their unambiguous success in preventing severe disease and deaths in infants and young children due to *B. pertussis*, improved aP vaccines are needed that provide longer (possibly life-long) protection, prevent low-grade infection and reduce transmission^82,83^. Natural pertussis infection does not convey life-long protection, and it is not yet certain if, or how, modified pertussis vaccines could achieve this goal. Adolescents originally primed with whole-cell pertussis vaccine demonstrate a longer duration of protection, probably linked to a Th1/Th17 priming response rather than the Th2-polarised or mixed Th1/Th2 responses induced by aluminium-adjuvanted aP vaccines^84^. Paediatric whole-cell-primed adolescents also respond more strongly to aP boosters as compared to paediatric aP-primed adolescents^85^. Natural infection induces the most durable immunity, possibly a result of imprinting of CD4+Th1 and Th17 cells that reside in the respiratory tract mucosal tissues^86^.

Improvements to aP vaccines could be achieved by increasing the content of antigen (pertussis toxin [PT] or fimbriae types 2 and 3 [FIM]), adding more antigens (such as adenylate cyclase), changing the adjuvant (toll-like receptor [TLR]-9 CpG adjuvant improved protection against pertussis compared to TLR4 adjuvant in mice injected with memory cells induced by aP^61^, suggesting that a paediatric Th2 imprint could possibly be reversed with a carefully selected booster formulation), or by changing the delivery system (for instance intranasal)^83^. All aP vaccines contain PT, a major virulence factor that requires detoxification before administration to humans. Most of the currently available aP vaccines use PT detoxified with formaldehyde and glutaraldehyde, resulting in preservation of approximately only 20% of surface epitopes^87^.

Genetic detoxification (PTgen) maintains the integrity of PT surface epitopes^87^, and PTgen has been available for decades^88–90^. The infant pertussis efficacy trials of the 1990’s included a PTgen vaccine that was more immunogenic than a chemically detoxified vaccine^91^. Head-to-head efficacy was similar but the chemically inactivated vaccine contained 5-times more PT (25µg vs 5µg). This PTgen-containing paediatric vaccine (Triacelluvax) was only marketed in Italy and was withdrawn from the market for commercial reasons in 2002 (linked to the need for large paediatric vaccine combinations such as diphtheria-tetanus-pertussis-hepatitis B-inactivated polio and Hib).The rather low duration of efficacy linked to adolescent Tdap booster immunisation may in part be explained by original antigenic sin, i.e., the imprinting of an immune response against non-protective epitopes linked to the chemical detoxification of PT, since chemically detoxified PT is the only available vaccine formulation in the vast majority of the world.

So far, the other major immune-evasion factor, the highly conserved adenylate-cyclase haemolysin toxin, has not been used in aP vaccines despite evidence of immune-protection^92,93^. As yet the potential contribution that detoxified adenylate-cyclase could make to aP vaccine efficacy is still being explored^93^.

### Lyme disease: withdrawal of an effective subunit vaccine

A subunit vaccine with 76% efficacy in preventing Lyme disease (*LYMERix*, GSK) was licensed in the US in 1998 but withdrawn in 2002^94,95^. While vaccines typically ‘fail’ due to concerns about efficacy or safety, the Lyme vaccine was withdrawn as a result of poor market performance, contributed to by weak recommendations, poor acceptance by the medical profession and patients due to lack of disease awareness, and adverse media coverage following unsubstantiated safety concerns^94,95^. In the case of Lyme disease prevention, further vaccine development is hindered not by specific challenges associated with the pathogen or the disease, but by perceived obstacles to its acceptance given past performance.

### Meningococcal serogroup B vaccines: in silico antigen discovery and subtractive antibody-screening

A polysaccharide-conjugate vaccine for MenB was ruled out due to poor immunogenicity of the MenB capsule and its similarity to human sialylated glycoproteins^96^. MenB subunit vaccines (rLP2086 and 4CMenB) were finally achieved using *in silico* antigen discovery and/or subtractive antibody-screening methods. Despite demonstrated efficacy, as yet the relative contribution of individual 4CMenB components to effectiveness is unclear^97^. Careful long-term evaluation of effectiveness and breakthrough strains is needed to resolve questions around the mechanisms of 4CMenB and rLP2086 protection, and to optimise vaccine composition if indicated.

## Progress in *S. aureus*, Expec, *S. pneumoniae* and tuberculosis vaccines

### Improving mucosal immune responses: adjuvants and alternative delivery systems

Adjuvants are immuno-stimulatory molecules used to enhance the immune response. The most recent adjuvants to be approved in human vaccines are AS01 (TLR4 agonist plus the saponin QS21) and CpG (TLR9 agonist)^36,37^. AS01 is included in the highly effective RZV but also in the experimental tuberculosis vaccine M72/AS01_E_, which showed statistically significant efficacy in preventing pulmonary tuberculosis in adults infected with Mtb^98^, suggesting that AS01 could be a suitable adjuvant for prevention of other mucosal infections. CpG was approved in a hepatitis B vaccine^37^.

The site at which dendritic cells encounter antigen determines the imprinting of homing receptors (tissue-specific adhesion and chemoattractant receptors) that control the migration of T- and B-cells to mucosal sites. Thus far, it has proven difficult to achieve high levels of T-cells at mucus membranes after systemic vaccination^99^. Alternative vaccination routes (oral, intranasal, sublingual, intra-vaginal, inhaled, transdermal) combined with adjuvants may have improved capacity to enhance T-cell homing, such as Trm cells, to specific mucosal sites^100^.

### *S. aureus*

Development of a *S. aureus* vaccine has proven far more difficult than anticipated. *S. aureus* excels in immune evasion and a series of vaccine failures using capsular polysaccharides and surface proteins clearly indicates that a successful vaccine will likely need to neutralise multiple key immune evasion factors^101,102^. *S. aureus* employs a host of diverse and redundant immune evasion mechanisms that derail the immune responses (B-and T-cell superantigens), inhibit recruitment of leucocytes, cause neutrophil lysis, interfere with complement activation, promote resistance to oxidative burst killing, provide innate resistance to antimicrobials, bind and degrade immunoglobulins, and form a protective fibrin capsule around the bacterium^103^. Which, and how many of these functions need to be inhibited to provide protection against diseases is not known, but the classical approach of using surface antigens (capsular conjugates or proteins) has thus far failed. The failed vaccine candidates from Nabi (CP5 and CP8 conjugates), Merck (IsdB) and Pfizer (CP5 and CP8 conjugates, ClfA and MntC) show common themes: a limited number of surface antigens, absence of immune-escape-related candidates, no use of adjuvants with failure to induce a CD4+ Th1/Th17 immune response, and a non-representative animal model (mice)^101,104,105^. Drawing on these failures, a successful strategy will require a multicomponent approach, possibly including immune-escape related candidate antigens, a better predictive animal model and addition of a Th1/Th17 adjuvant to enhance the immune response^51^. For the activation and recruitment of macrophages and neutrophils, Th1 (IFN-y) and Th17 (IL-17A) cells are essential^106^. Genetic deficiencies in phagocyte function, Th17 and IL-17RA all predispose to staphylococcal infections, confirming the mechanisms described above^60,107^.

### *ExPEC* and *S. pneumoniae* mucosal diseases

ExPEC is the most common cause of UTI. Initial results from Phase 1–2 studies suggest that a systemic vaccine to prevent UTI may be feasible. One study has explored a prototype 4-valent ExPEC bioconjugate vaccine in women with a history of recurrent UTI^45^. Another has assessed a vaccine containing FimH from type 1 fimbriae and a new adjuvant (https://sequoiasciences.com/uti-vaccine-program). ExPEC conjugate vaccines have a good chance to prevent invasive ExPEC disease (predominantly bacteraemia and urosepsis) but would likely need to be enhanced to optimally induce mucosal immunity. As for other conjugate vaccines however, modification of the formulation to induce a Th1/Th17 response may help to achieve clinically relevant efficacy against UTI. Such an approach could also be used to increase the efficacy of PCVs against non-bacteraemic pneumonia^108^, although achieving a correctly balanced response is proving challenging. A candidate pneumococcal vaccine comprised of three proteins with Th1/Th17-inducing capacity (SP0148, SP1912, SP2108), but with Th2-inducing aluminium hydroxide as adjuvant, failed to show efficacy after human challenge for pneumococcal colonisation^109^. Another protein pneumococcal candidate vaccine using aluminium phosphate also failed to prevent colonisation^110^, whereas a protein vaccine using a potent Th1 adjuvant protected non-human primates from pneumonia^76^.

### Tuberculosis

*M. tuberculosis* is an intracellular bacterium and its lipoidal surface structure further complicates the options for immune attack. After infection, aggregation of macrophages and monocytes and the presence of chemokines that promote cell adhesion and T-cell recruitment underlie the formation of organised structures called granuloma, often leading to a long latent phase of the infection^41^.

The Bacille Calmette-Guerin (BCG) vaccine has been in continual use since the 1960s and is effective in preventing disseminated tuberculosis in children. BCG appears to accelerate Mtb dissemination from alveolar macrophages to tissue-recruited or lung-recruited macrophages and neutrophils via antigen-specific CD4 T-cells^111^. This transfer to phagocytes and their activation and differentiation is thought to increase the ability to control bacterial replication through as yet unknown mechanisms^111^. BCG induces trained innate immunity which enhances the ability of innate effector cells to respond to non-specific stimuli^112,113^.

Re-activation of disease may circumvent this transfer step, which could contribute to the lower effectiveness of BCG in preventing pulmonary re-activation in infected adults, which is the major means by which tuberculosis is transmitted and the most common cause of tuberculosis-related deaths^114,115^.

The protective adaptive response is thought to be mediated mainly by CD4+ Th1 cells with contributions from Th17 and CD8+ T-cells, but as yet no solid explanation how bacterial killing is finally achieved is forthcoming^116,117^. Recent findings from India using BCG revaccination in IGRA+ (IFN-y release assay linked to presence of Mycobacteria) subjects demonstrated boosting of Th1/Th17 CD4+ T-cell responses including polyfunctional T-cells^65^.

BCG prevented sustained seroconversion (by IFN-γ release assay) in South African adolescents when given as a booster^66^. Newly developed live-attenuated mycobacterial vaccines (MTBVAC; VPM1002) are being evaluated as potential replacements/improvements for BCG^17^.

While the immunology of CD4+ responses in tuberculosis has received considerable attention^117^, there has been less focus on the role of bacterial virulence mechanisms. Incomplete knowledge of Mtb secretory systems and bacterial defenses against reactive oxygen and nitrogen molecules and ill-defined mechanisms of bacterial virulence and immune protection are major obstacles in the quest for an improved tuberculosis vaccine, and have made selection of target antigens for vaccine inclusion problematic. The 11 tuberculosis vaccine candidates currently in clinical development include killed whole-cell, live attenuated, adjuvanted subunit and viral-vectored designs (reviewed recently^117^). Several vaccines (MIP, Vaccae, H56:ICI31, RUTI™, and ID93/GLA-SE) are being developed as adjuncts to tuberculosis treatment. Development of therapeutic vaccines to be used in integrated approaches with antimicrobial treatment could proceed rapidly given the relative ease of detecting clinical endpoints and may be a ‘quick win’ in terms of increasing success rates in the treatment of MDR tuberculosis.

Four adjuvanted subunit vaccines are in clinical evaluation. These contain proteins whose function and relationship to virulence is not always known (Table 1). All four protein subunit vaccines and two viral-vectored vaccines (AERAS-402 and MVA85A) induced similar functional Th1 CD4+ T-cell responses, without significant CD8+ T-cells or IL-17 production, the only differentiation being higher cytokine responses in recipients of M72/AS01_E_ (GSK), a recombinant fusion protein with AS01^118^. A prime-boost schedule using the viral-vectored candidate vaccines AERAS-402 and MVA85A found that the booster dose enhanced CD8+ T-cell responses but that these were short-lived^119^.

**Table 1:** Candidate vaccines targeting priority pathogens in clinical development.

Vaccine	Antigen composition	Function	Immune stimulatory mechanism	Desired vaccine response	Phase
***S. pneumoniae***					
20vPCV (Pfizer)	Capsular polysaccharide	Capsular polysaccharide virulence factor - 20 serotypes	Protein conjugation	T-cell dependent induction of memory B cells and opsonophagocytic antibodies	3
ASP3772 (Astellas Pharma Inc)	Multiple components Multiple Antigen-Presenting System	Unknown	Unknown	Th1/Th17 responses	1/2
V114 (Merck)	Capsular polysaccharide	Capsular polysaccharide virulence factor - 15 serotypes	Protein conjugation	T-cell dependent induction of memory B cells and opsonophagocytic antibodies	3
Unnamed (SutroVax)	Capsular polysaccharide	Capsular polysaccharide virulence factor - 24 serotypes	Protein conjugation	T-cell dependent induction of memory B cells and opsonophagocytic antibodies	1 anticipated
***S. aureus***					
rTSST-1 (Biomed)	Detoxified double mutant Toxic shock syndrome toxin-1	Major virulence factor. Causes endotoxic shock	Aluminium adjuvant	Th17 response. Neutralising antibodies	2
STEBVax (IBntegrated Biotherapeutics, NIAID)	Enterotoxin B	Major virulence factor. Causes endotoxic shock	Aluminium adjuvant	Neutralising antibodies	1
NDV-3 (NovaDigm)	Als3	Invasive like protein of *Candida albicans* required for ferritin binding	Aluminium adjuvant	Antibody and T-cell responses	2
Unnamed (Chengdu Olymvax Biopharmaceuticals Inc)	Unknown		Unknown	Unknown	2
***B. pertussis***					
GamLPV (GRIEM, HMRFHealth Ministry of the Russian Federation)	Live-attenuated	Transient colonisation of the human airway	Intranasal administration	Unknown	1/2
BPZE1 (NIAID)	Live-attenuated	Transient colonization of the human airway	Intranasal administration	IL-17 responses	2
***E. coli***					
ExPEC10V (Janssen Research & Development, LLC)	Conjugated O-serotypes	Capsular polysaccharide virulence factor – multiple O-serotypes	Protein bioconjugation	T-cell dependent induction of memory B cells and opsonophagocytic antibodies	1/2
Unnamed UTI vaccine (Sequoia Sciences)	FimCH	Fimbrial adhesin protein needed for epithelial attachment	Adjuvant	Unknown	1
***M. tuberculosis*** **subunit vaccines**					
ID93/GLA-SE (IDRI Wellcome Trust, Quratis)	Rv1813	Unknown. Possibly a secreted protein that is up-regulated during hypoxia or dormancy antigen	GLA-SE adjuvant	TLR4 agonist induces a Th1 response	
	Rv2608	PPE 42 protein			
	Rv3619	EsAT6 virulence factor expressed continually during infection			
	Rv3620	EsAT6 virulence factor expressed continually during infection			2
M72/AS01_E_ (GSK)	Mtb39A (Rv1196)	Membrane-associated PPE 18 expressed early in infection. Genetic variations exist	AS01_E_ adjuvant	TLR4 agonist induces a Th1 response	2
	Mtb32A (Rv0125)	Secreted protein possible serine protease			
H56:ICI31 (SSI, Valneva, Aeras)	Ag85B (Rv1886c)	Mycolyl transferase involved in cell wall synthesis	Valneva IC31© adjuvant	TLR9 agonist	2
	Rv3875	EsAT6 virulence factor expressed continually during infection			
	Rv2660c	Possible stress-induced or dormancy antigen associated with latent disease			
H4:IC31 (SSI, Valneva, Aeras)	Ag85B (Rv1886c)	Mycolyl transferase involved in cell wall synthesis and host T-cell entry	Valneva IC31© adjuvant	TLR9 agonist induces a Th1 response	2
	TB10.4 (Rv0288)	ESX family of secretory proteins			

*IB* Integrated Biotherapeutics, *GRIEM* Gamaleya Research Institute of Epidemiology and Microbiology, *HMRF* Health Ministry of the Russian Federation, *IDRI* Infectious Disease Research Institute, *MAPD* multiple antigen-presenting system, *NIAID* National Institute of Allergy and Infectious Diseases, *PPE* abundant family of proteins with conserved Pro-Pro-Glu (PPE) motifs at the N terminus, *SSI* Statens Serum Institute

After 2-years of follow-up, vaccination with M72/AS01_E_ provided 54.0% protection (90% CI 13.9–75.4) against active pulmonary tuberculosis disease in infected adults in South Africa, Kenya and Zambia^120^. Vaccine efficacy after 3 years was 49.7% (90% CI 12.1–71.2)^98^.

H56:IC31, comprising a fusion protein and IC31 adjuvant (Table 1) is being developed to prevent tuberculosis disease in infected individuals of all age-groups and as an adjunct to antimicrobial treatment. This vaccine candidate induces antigen-specific Th1 immune responses in infected and uninfected adults^121^, and is being evaluated in two Phase 2 studies for efficacy as adjunctive treatment (NCT02503839, NCT03512249).

As yet, subunit vaccines include a limited set of antigens whose individual protective abilities are largely unknown^122^. A renewed systemic full-genome based antigen discovery effort is needed to add greater certainty to antigen selection. Efforts are underway with extended subunit compositions being investigated as DNA or viral (cytomegalovirus)-vectored vaccines^123,124^.

## Outlook

The primary challenges for bacterial vaccines are to address the global problem of AMR, to address diseases of world-wide significance, and to expand into a life-course strategy to meet the specific needs of seniors. An important segment of the AMR challenge, and beyond the scope of the present review, is the role of veterinary bacterial vaccines to reduce antimicrobial use in the veterinary sector and zoonotic infections to prevent transfer of resistance genes/plasmids to the human bacterial pool (recently reviewed in ref. ^125^).

Significant progress in human bacterial vaccine development is being made and there is reason to believe that reductions in MDR infections and effective prevention of disease in seniors is achievable. Development of a senior vaccine ‘package’ is starting to take shape with influenza, pneumococcal and herpes zoster vaccines. The high efficacy of RZV shows that overcoming immune senescence can be achieved by using appropriate adjuvants. Bioconjugate vaccines under development targeting invasive ExPEC disease are potentially the next member of the senior franchise and will also be critical for the prevention of MDR ExPEC infections, while intensive efforts continue on vaccines for *S. aureus* and improved pneumococcal vaccines. Combining neutralising antibodies with bystander CD4+ T-cell responses that includes Th1 and Th17 responses deserves exploration for the prevention of mucosal diseases such as UTI, pneumonia and acute bacterial skin/skin structure infections. This approach may lead to a new wave of successful bacterial vaccines. Vaccines targeting other ESKAPE pathogens and *C. difficile* are currently in discovery and early development phases. Over the last century bacterial vaccines have saved countless lives and their role is set to expand over the next century in response to the specific needs arising from MDR and ageing populations. While vaccine advances are crucial, averting a crisis in bacterial disease must be accompanied by the discovery of targeted alternatives to broad-spectrum antimicrobials, rapid bedside diagnostic tests of antimicrobial susceptibility, and responsible antimicrobial stewardship.
